# All‐Optical Modulation Photodetectors Based on the CdS/Graphene/Ge Sandwich Structures for Integrated Sensing‐Computing

**DOI:** 10.1002/advs.202413662

**Published:** 2025-01-22

**Authors:** Qi Yang, Jie Hu, Haozhou Li, Qing Du, Shuanglong Feng, Dong Yang, Yupeng Zhang, Jun Shen

**Affiliations:** ^1^ Chongqing Institute of Green and Intelligent Technology Chinese Academy of Sciences Chongqing 400714 P. R. China; ^2^ College of Electronics and Information Engineering Shenzhen University Shenzhen 518060 P. R. China

**Keywords:** all‐optical modulation, integrated sensing‐computing, moving object recognition, photodetector, ultra‐wide response range

## Abstract

In this manuscript, an all‐optical modulation photodetector based on a CdS/graphene/Ge sandwich structure is designed. In the presence of the modulation (near‐infrared) light, the Fermi level of the graphene channel shifts, allowing for the tuning of the visible light response speed as well as achieving a broad responsivity range from negative (‐3376 A/W) to positive (3584 A/W) response. Based on this, logical operations are performed by adjusting the power of the modulation light superimposed with the signal light. This facilitates more covert, all‐optical, high‐speed encrypted communication. The ultrahigh tunability and nearly symmetric positive and negative photoconductivity of all‐optical modulation photodetectors significantly enhance the computational capacity of neuromorphic hardware. The proposed device exhibits substantial advantages in applications requiring high fault tolerance for integrated sensing‐computing （ISC） and high‐resolution motion object recognition, providing insights for the development of next‐generation high‐bandwidth, low‐power‐consumption ISC devices.

## Graphical abstract

This study designs an all‐optical modulation photodetector based on a CdS/graphene/Ge sandwich structure. Modulation with near‐infrared light enables tunable visible light response, achieving a wide responsivity range (‐3376 to 3584 A/W). Logical operations and encrypted communication are realized, enhancing neuromorphic computing capacity. The device offers promising applications in high‐bandwidth, low‐power integrated sensing‐computing and motion object recognition.

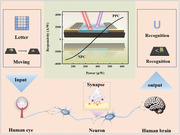

## 1 Introduction

The evolution of photodetectors has evolved from focusing solely on high‐performance visual detection to encompassing multifunctionality.^[^ ^1^, ^2^, ^3^ ^]^ For instance, dual‐band photodetectors^[^ ^4^, ^5^ ^]^ improve identification accuracy by simultaneously detecting two wavelength bands. Integrated sensing‐computation photodetectors (ISC PDs) combine visual perception with pre‐computational processing.^[^ ^6^, ^7^, ^8^ ^]^ The latter, commonly known as the electrically modulated ISC PDs feature a gate that modulates the channel of the photoconductive detector, enabling both positive and negative photoconductance (PPC and NPC). These properties mimic the excitatory and inhibitory signals in retinal neural systems, facilitating brain‐like computation.^[^ ^9^, ^10^, ^11^ ^]^ However, electrically modulated devices often require complex circuit designs and control logic, and are constrained by low bandwidth and poor electromagnetic interference resistance. In contrast, all‐optical modulation ISC PDs^[^ ^12^, ^13^, ^14^, ^15^ ^]^ offer significant advantages, including simplicity of structure, high bandwidth, and superior interference resistance.^[^ ^16^, ^17^, ^18^, ^19^, ^20^ ^]^ Furthermore, by eliminating reliance on electrical signals, all‐optical modulation ISC PDs are more suitable for the construction of neuromorphic systems.^[^ ^21^, ^22^, ^23^ ^]^

Existing all‐optical modulation ISC PDs face two major challenges. On the one hand, although some studies have demonstrated the ability to achieve both positive and negative bidirectional all‐optical modulation by altering the wavelengths of different incident lights,^[^ ^4^, ^24^, ^25^, ^26^, ^27^ ^]^ this approach frees the devices from the constraints of electrical signal and is more conducive to the development of artificial vision systems. However, these studies achieve bidirectional modulation by replacing the incident laser with different wavelengths. Since the replacement process is discontinuous, it cannot achieve synchronous modulation in the temporal dimension compared to the method that utilize continuous variations of parameters using a single wavelength of light, which significantly limits their application range.^[^ ^28^ ^]^ On the other hand, the computational scale of ISC PDs is often closely related to the size of the employed computational matrix. When the same computational matrix is employed, for example, a 3 × 3 matrix is used, a broader positive and negative response range of the ISC PDs can maintain a greater degree of discreteness between the filling values, thereby reducing the recognition error rate of the corresponding signal at each matrix cell and effectively decreasing the recognition misjudgment rate in the final recognition outcome. If the matrix adopts the same degree of discreteness for the filling values and the misjudgment rate remains the same, a broader responsivity range allows for the employment of a larger matrix to enhance the recognition resolution and obtain more detailed information. However, due to factors such as material band alignment and interfacial treatment methods, the adjustable responsivity range in most current studies is relatively narrow, and there is a widespread asymmetry in the positive and negative responsivity ranges.^[^ ^28^, ^29^, ^30^ ^]^ Furthermore, the asymmetry of PPC and NPC in certain applications that rely on positive and negative symmetric values for calculations, such as motion recognition scenarios, limits the usable range to the smaller absolute value, further restricting the resolution in these contexts. In light of these challenges, developing all‐optical bidirectional modulation ISC PDs with continuous tunability, an ultra‐wide modulation range, and symmetric PPC and NPC will enhance computing power, enable more secure encrypted communication, and facilitate higher‐resolution ISC devices, which holds significant research potential.

In this work, we propose the all‐optical modulated ISC PDs based on a CdS/graphene/Ge sandwich structure. The fabrication processes of the two heterojunctions were conducted in an oxygen‐free environment. This approach effectively minimized interface defects caused by oxide layers and adsorbed small molecules, such as oxygen, thereby improving the interface quality. However, the inevitable adsorption of small molecules, such as water, ultimately rendered the graphene p‐type. By simply altering the modulation light power, the CdS/graphene and graphene/Ge heterojunctions regulate the Fermi level of graphene through the photogating effect, enabling the device to transition from NPC to PPC. This transition facilitates excitatory and inhibitory synaptic behavior within the optical pathway. Notably, the ingenious band alignment and selection of modulation power results in symmetric PPC and NPC values. We developed a dark‐code‐based signal encryption method based on the device's power‐induced photoconductivity transition characteristics. Additionally, the device's bidirectional tunable photoresponse and ultra‐wide tunable range were employed to achieve image perception, recognition, and processing, demonstrating its application in ISC systems, such as moving object recognition.

## 2 Results and Discussion

### 2.1 Design Principle and Device Performance

To address the limitations which inhibit their application in high‐resolution systems and encrypted communications of existing all‐optical modulation ISC PDs, especially the narrow modulation range and the asymmetry of PPC and NPCasymmetric of positive and negative photoresponses, we propose a photodetector based on a CdS/graphene/Ge sandwich structure. Our analysis reveals that the primary factors contributing to the poor quality of the heterojunction interface arise from the adsorption of small molecules, such as oxygen from the air, onto the graphene when it forms van der Waals heterostructures with other materials. Additionally, the natural oxidation layer on the surface of substrates like Ge further increases the concentration of carrier traps at the heterojunction interface, thereby hindering carrier transport across the interface.

**Figure 1:**
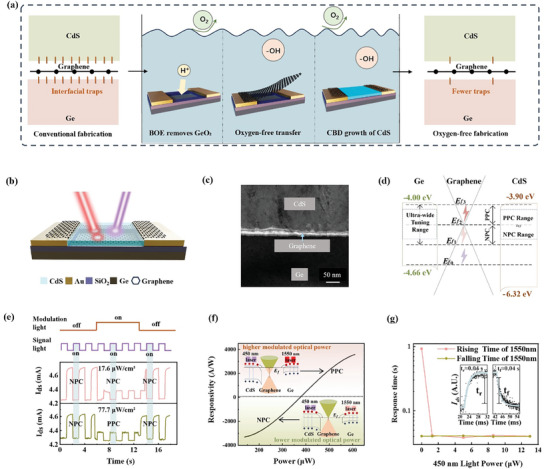
Interface‐optimized all‐optical modulation device and its PPC and NPC mechanisms and performance. a) Schematic diagram of the oxygen‐free fabrication process of sandwich heterojunctions. b) Schematic of the all‐optical modulation detector structure. c) TEM cross sectional image of the device's heterojunction interface. d) Mechanism of PPC and NPC in the device. e) Transition from NPC to PPC under varying modulation light power densities. f) Photoconductivity response of the device as a function of modulation light power. g) ISC PDs possess the capability for response time modulation.

To mitigate this issue, first, we employed a buffered oxide etchant (BOE) solution to remove the oxide layer on the Ge surface prior to transferring the graphene. After rinsing it in a wet state, we utilized a wet‐transfer^[^ ^31^, ^32^, ^33^ ^]^ method to deposit the graphene, thereby avoiding damage to the graphene/Ge heterojunction caused by oxide layers and small molecules like oxygen.^[^ ^34^, ^35^ ^]^ Second, to ensure high‐quality fabrication of the CdS/graphene heterojunction, we employed the chemical bath deposition (CBD) method to grow CdS conformally layer by layer on the graphene surface in an oxygen‐free aqueous environment, preventing the adsorption of small molecules on the surfaces of graphene. These adsorbates can capture electrons from the graphene, forming negatively charged oxygen ions and inducing a pinning effect on the graphene energy level, thus limiting the tunable range of the graphene's Fermi level. The final oxygen‐free sandwich heterojunction interface with fewer defects, as shown in **Figure** **1**a, thereby laying the foundation for an extended modulation range of responsivity.

With these core process improvements, the device structure was fabricated as shown in Figure 1b, and the transmission electron microscopy （TEM） and scanning electronmicroscopy （SEM） images of the device are shown in Figure 1c and Figure S1 (Supporting Information). Raman characterization of graphene, along with the thickness and absorbance characterizations of different materials collectively validate our conclusions, as detailed in the Supporting Information and Figures S2‐S4. In the sandwich‐structured device, high‐mobility graphene serves as the conductive channel for the photoconductive detector, while Ge and CdS act as the primary light‐absorbing layers. Due to Ge's smaller bandgap compared to CdS, the Ge/graphene heterojunction can detect longer‐wavelength signals (580 nm < λ <1850 nm), while the CdS/graphene junction detects shorter‐wavelength signals (λ < 580 nm). After optimizing the material thickness, we achieved selective absorption and detection across both wavelength bands.

Following these preparations, we first verified the all‐optical modulation detection performance of the dual‐band responsive device (Figure S5, Supporting Information). By varying the power of the modulation light, the all‐optical modulation detector exhibited various responses, including PPC, NPC, and zero photocurrent, mimicking fundamental synaptic behaviors such as excitation and inhibition.

We provide the following theoretical explanation for the ultra‐wide and symmetric modulation range from the perspectives of the device's energy band positions and the optical modulation mechanism as follows. As illustrated in Figure 1d, given that CdS has a larger bandgap compared to Ge, the highest valence band value is determined by Ge, and the conduction bands of both materials differ by only 0.1 eV. Consequently, the tunable range of the graphene energy level^[^ ^36^, ^37^ ^]^ is minimally affected by band confinement, explaining the broad modulation range. Furthermore, the hydroxylated, oxygen‐free environment employed in the fabrication of the heterojunction significantly reduces interface adsorbates and defects, thereby mitigating the pinning effect and enhancing the modulation range. Since the PPC corresponds to the *E_f_*_3_ *∼ E_f_*_2_ range and the NPC corresponds to the *E_f_*_2_ ∼ *E_f_*_1_ range, the device exhibits a relatively symmetric modulation range. The results of the Sentaurus‐TCAD simulations, which modeled the internal electric field distribution under illumination, as shown in Figure S6 (Supporting Information), further corroborate our explanation of the Fermi level shifts under illumination.

We systematically analyzed the optoelectronic detection performance of the CdS/graphene/Ge detector, supported by the band modulation theory underlying PPC and NPC. We first verified the NPC and PPC transitions using lower and higher 1550 nm modulation light powers, respectively, as demonstrated by the device's I‐T curves shown in Figure 1e. The transition nodes and processes are depicted in Figure S7 (Supporting Information). To confirm that the transition process involves electron injection into the graphene channel, we designed a device and conducted transconductance tests for verification, as shown in Figure S8 (Supporting Information).

Based on the measured photocurrent, we calculated and fitted the responsivity variation curves, as shown in Figure 1f. In the NPC regime, the net photocurrent decreases with increasing modulation light power, achieving a maximum responsivity of ‐3376 A/W. This high responsivity is attributed to the photogating effect, where photogenerated carriers undergo spatial separation, extending the lifetime of one carrier type, resulting in ultra‐high gain and responsivity. In the PPC regime, the net photocurrent increases with increasing modulation light power, with a maximum responsivity of 3584 A/W. To demonstrate the continuous bidirectional modulation capability of this study and the technical advantages of the ultra‐large and highly symmetric PPC and NPC modulation ranges, we have compared recent all‐optical bidirectional modulation devices, as shown in **Table** **1**.

**Table 1:** Comparison of bidirectional modulation methods, responsivity ranges, and symmetry of PPC and NPC for different all‐optical bidirectional modulation devices.

Active layer	Bidirectional modulation method	Responsivity [A/W]	Symmetry of PPC and NPC	References
graphene/C60/pentacene	changing laser wavelengths	R_PPC_ = 7673 R_NPC_ ∼ NG	–	[12]
MoS_2_/ graphene/Ge	changing laser wavelengths	R_NPC_ = 1.1 R_PPC_ = 8	No	[16]
In_2_O_3_/Al_2_O_3_/Y6	changing laser wavelengths	R_NPC_ = 0.1 R_PPC_ = 0.38	No	[22]
Graphene/TiO_2_QD	changing laser wavelengths	NG	–	[24]
CuPc/P(VDF‐TrFE)	changing laser wavelengths	NG	–	[26]
black phosphorus	changing laser wavelengths	NG	–	[27]
Bi_2_O_2_Se/graphene	changing laser wavelengths	R_NPC_ = 110 R_PPC_ = 88	No	[29]
graphene/InSe/*h*‐BN	different powers of a single laser	R_NPC_ = 1.1 × 10^4^ R_PPC_ = 13	No	[28]
CdS/graphene/Ge	different powers of a single laser	R_NPC_ = 3376 R_PPC_ = 3584	Yes	This work

Additionally, we found that the introduction of modulation light significantly improves the device's response speed. As shown in Figure 1g, under 1550 nm illumination alone, the rise time is 0.92 s. However, when illuminated simultaneously with 450 and 1550 nm, the rise time stabilizes at ≈0.04 s, regardless of the 450 nm power level. The fall time of PDs at a wavelength of 450 nm is largely unaffected by light with a wavelength of 1550 nm. The theoretical explanation for this phenomenon is provided in the supplementary information under section 2.

### 2.2 Single‐Beam Light Modulation for Encrypted Communication Using Logic Operations

Under the modulation of 1550 nm infrared light, the Fermi level of graphene changes during the process of electron injection and recombination. However, within a certain range of modulation light power, this change tends to stabilize, as reflected in the small fluctuations and consistent trend of the device's photocurrent. Utilizing this stability, we can leverage the bipolar response characteristics of the Ge/Graphene/CdS‐based optical modulation for logic encryption coding and signal transmission. For instance, when 450 nm light is used as the periodically switched signal, varying powers of 1550 nm light will generate corresponding PPC and NPC, and the tunable range of the photocurrent is relatively large, indicating the potential for multi‐state output. Notably, unlike other studies that utilize different wavelengths of incident light to separately achieve PPC and NPC, we employ two wavelengths simultaneously while only altering the modulated light power to correspond to “0” and “1” encoding. This approach significantly enhances the security of encrypted communication, as it becomes exceedingly difficult to decipher the encoded values based solely on intercepted wavelength information.

**Figure 2:**
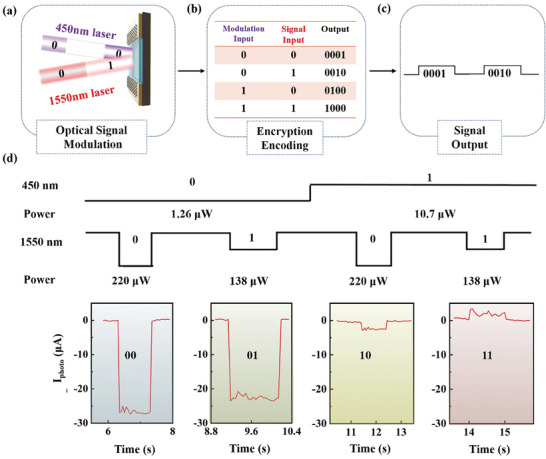
Single‐beam modulation light for signal light, achieving all‐optical logic encryption operations. a) Various power signals and modulation light are encoded. b) Preset codebook for the combination of signal light and modulation light. c) Output encrypted logic signals. d) By selecting two power levels of 450 nm and 1550 nm, four types of logic signal outputs can be achieved.

By simultaneously applying two light beams and encoding based on different power levels, we can establish corresponding codebooks using combination methods. If we encode the two power levels of light from two wavelengths as “0” and “1” respectively, as shown in **Figure** **2**a, we can perform secondary encryption on the combination forms 00, 01, 10, 11 by setting a predetermined codebook, corresponding to photocurrent outputs of 0001, 0010, 0100, 1000, as shown in Figure 2b,c. This approach enables us to achieve logic operations and all‐optical encrypted communication. Specifically, we consider a 450 nm light power of 1.26 and 10.7 µW corresponding to 0 and 1, respectively, and a 1550 nm light power of 220 and 138 µW corresponding to 0 and 1 (Figure 2d). Different combinations correspond to four two‐bit combination outputs: 00, 01, 10, and 11. If more power levels are utilized as inputs, the number of decoding bits can be further expanded.

### 2.3 Application of Matrix Modulated Light in Integrated Sensing‐Computing Systems and Moving Object Recognition

For the proposed CdS/Graphene/Ge all‐optical modulator, its tunable positive and negative photoresponse allows it to simulate excitatory and inhibitory synaptic behaviors within the optical path.^[^ ^38^ ^]^ The photoresponse depends on the modulation light power, allowing for different convolution kernel configurations by adjusting the modulation light power, thereby performing various operations on the image. As a proof of concept, we used a single device to sequentially receive pixel images for convolution processing, as illustrated in Figure S9 (Supporting Information). We simulated Gaussian blur operation using a 3 × 3 array and set the device to three different photoresponse states. By adjusting the modulation light power, the 3 × 3 Gaussian operator $\left(\begin{array}{ccc} 6.3 & 12.0 & 6.3\\ 12.0 & 16.5 & 12.0\\ 6.3 & 12.0 & 6.3 \end{array}\right)$ and its weights were mapped to the responsivity of the CdS/Graphene/Ge optoelectronic ISC PDs, forming the corresponding convolution kernel. The distribution of photoresponse states across different devices is shown in Figure S10 (Supporting Information). Image processing was conducted using a single‐layer perceptron neural network and the matrix multiplication formula *I = ∑ P×R*. The grayscale value was reflected in different signal light power intensities, with network weights applied to multiple photoresponse states.

We processed the logo of the Chongqing Institute of Green and Intelligent Technology, Chinese Academy of Sciences, using various convolution kernels, including Gaussian, Roberts‐x, Roberts‐y, Prewitt‐x, and Prewitt‐y. The results demonstrated good performance across these different image processing methods. Leveraging the computational capability of the described device, additional image processing operations such as inversion, blurring, embossing, sharpening, and edge extraction can also be performed. These functions are easily achieved by customizing the modulation light power encoding.

**Figure 3:**
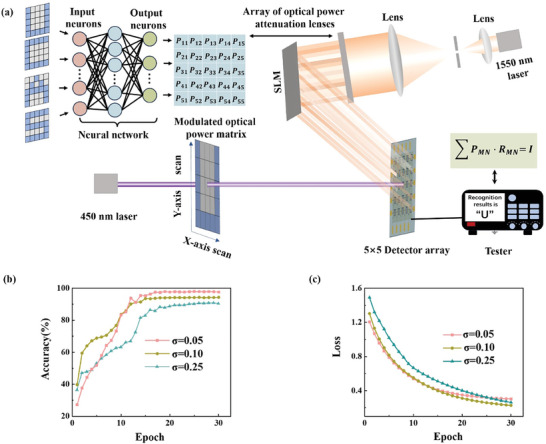
Image perception‐processing integration using matrix modulated light in all‐optical modulation ISC PDs. a) A convolutional neural network (CNN) was trained using the letters “UCAS” to derive a pre‐processing modulated optical power matrix, which is then used for alphabet recognition. b,c) Post 30 training epochs, the recognition accuracy under varying Gaussian noise variances and the loss function values are presented.

Building upon the foundation of visual image processing, the integration of ISC PD's PPC and NPC capabilities allows for the further expansion of its applications in visual signal detection and preprocessing. By leveraging convolutional neural networks, these advancements facilitate the development of image recognition systems.^[^ ^39^, ^40^, ^41^ ^]^ As illustrated in **Figure** **3**, we present an optoelectronic sensor array that functions as an artificial neural network, capable of simultaneously sensing and processing projected images. The sensor employs a light response matrix to perform real‐time multiplication operations on the projected image. Training the network involves setting the light response values for each pixel individually. The modulation light power applied to each pixel updates the weights. Using Kirchhoff's law, the total output current after multiplying the perceived image information with each pixel's light response weight is calculated. By employing backpropagation, we adjust the modulation light power at each pixel to update the weights after each epoch. Compared to traditional systems based on von Neumann architecture and silicon CMOS technology, neuromorphic vision systems based on artificial neural networks have demonstrated satisfactory results^[^ ^35^, ^36^, ^37^ ^]^ in efficiently processing visual information, akin to the human brain's visual cortex, while reducing hardware complexity and power consumption.

In neuromorphic vision systems, the sensor array mimics the perception and recognition functions of the human retina. Images are transferred to a three‐layer artificial neural network for training and recognition, as depicted in Figure 3a. This neural network consists of input, hidden, and output layers for image recognition. The training dataset comprises 5 × 5 grayscale images representing the letters “U”, “C”, “A”, and “S”, formed by blue squares, with random noise of varying sizes and gray levels. These letter‐shaped instructions are input into the sensor via signal light scanning, generating a random light response matrix. The light power modulation for each pixel depends on the measured photocurrent feedback from the input image.

In the trained neural network, the category information of the input image is computed using the following formula:

(1) $$I_{k}=\sum_{i.j}^{m,n}R_{i,j}^{k}\;P_{i,j}$$

where *I_k_* is the output current vector, $R_{i,j}^{k}$ is the responsivity matrix, and *P*_*i*,*j*_ is the light image vector projected onto the detector. Through continuous updates of the light response values using the backpropagation algorithm, recognition accuracy improves. The extracted weight values *R* adjust the light response matrix to match specific input images. This process involves modifying the light response matrix to minimize the error between the output current and the expected feature current. The trained weight values *R* are distributed to the sensor array via a tuning device. The allocated detectors receive input images and output specific currents, enabling image recognition through feature currents.

As shown in Figure 3b,c, the 2000 samples after 20 training epochs under different noise variance levels, the recognition accuracy exceeds 90%, and the loss function values (indicating the deviation between the model predictions and training target expectations) significantly decrease, suggesting that model predictions closely align with reality. The trained modulation light matrix, transformed into a 5 × 5 light power matrix through an optical path, multiplies with the row‐by‐row scanned recognition samples (5 × 5 chromaticity matrices of UCAS letters with varying noise levels), projecting onto the 5 × 5 device matrix. The total device current is then used to sense and compute the recognized letter from the input.

**Figure 4:**
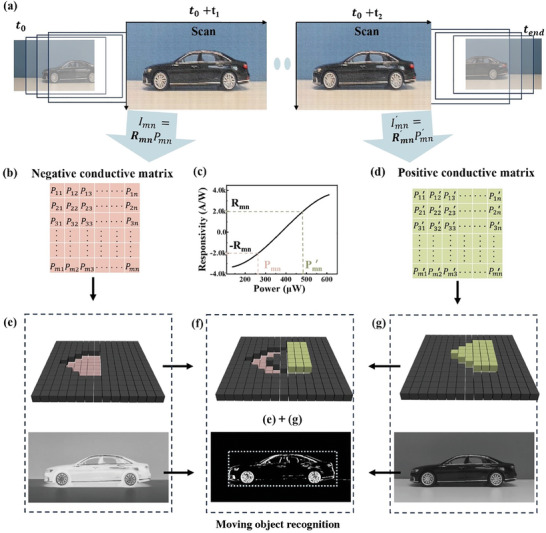
Moving object recognition in continuous frames using an all‐optical modulator. a) ISC PDs use a line‐by‐line scanning of arbitrary two video frames as the signal light. b–d) Pre‐set two modulation light power matrices with equal absolute values of responsivity but opposite in sign according to the device's response characteristics. e–g) The image is constructed by summing the results of two output current matrices, which facilitates the identification of object motion trajectories.

By expanding the input signal light matrix size and utilizing positive and negative light response devices to design a responsivity cancellation matrix, the all‐optical modulation device can recognize moving objects. With the advent of the Internet of Things (IoT) era, the detection and recognition of moving targets have become increasingly important.^[^ ^42^ ^]^ Currently, motion detection and recognition (MDR)^[^ ^43^ ^]^ technology based on CMOS image sensor^[^ ^44^ ^]^ platforms involves redundant sensing, transmission conversion, processing, and storage modules, making existing systems bulky and inefficient. In this study, by inducing optical stimulation to generate adjustable positive and negative light responses combined with inter‐frame differential calculations, motion target detection and recognition can be achieved. **Figure** **4** illustrates the working principle of moving object recognition. By combining two modulation light power matrices with equal magnitudes and opposite signs, motion detection and recognition functions can be realized. A motion process video can be subdivided into a series of images at different times from t_0_ to t_end_. We use a positive response matrix to detect the image information of the frame at t_0_ and a negative light response matrix to detect the image information of the frame at tend. The array's output photocurrent follows the rule of Formula 1, which is the product of the light response and the input laser power. When combining the photocurrents of the two arrays, the pixel photocurrents responding to static object images cancel each other out due to their opposite signs. Since the response amplitudes of the positive and negative responses are similar, the total photocurrent sum approaches zero, making it easy to obtain a resulting frame containing only moving object information. If no moving objects are present in the individual frames, each pixel remains dark as the photocurrents with opposite signs cancel each other out. The sensor array can detect moving objects through the all‐optical modulation detector, significantly reducing the transmission and interaction of various information compared to traditional motion detection technology. Notably, its structure is similar to that of the human retina, with the potential to achieve multiple eye functions by modifying and superimposing optical information in both time and amplitude. Internal processing within the sensor is expected to reduce energy consumption and hardware complexity for moving object recognition.

## 3 Conclusion

In summary, we propose an all‐optical modulation detector featuring a CdS/graphene/Ge sandwich structure. By modulating the Fermi level of graphene with incident light of varying wavelengths, the device exhibits both positive and negative responses. Initially, we employed dual‐beam light for more discreet multi‐digit encrypted communication compared to single‐beam light. We further upgraded the single‐beam modulation light to a 5 × 5 modulation light power matrix trained via a CNN, thereby achieving excitatory and inhibitory synaptic behaviors in the optical path for ISC recognition. Additionally, we make full use of the high symmetry of PPC and NPC and realized recognition of moving objects by using a cancellation matrix as the modulation light power matrix, thereby validating the feasibility of integrated sensing‐computing at the device level and broadening the application scope of ISC PDs. The ultra‐wide tunable responsivity range demonstrated in this study provides a greater selectable threshold for improving matrix network performance. This significantly expands the processing and computational capacity of neuromorphic hardware while enhancing computational stability through a reasonably increase in threshold spacing, thereby offering new developmental perspectives for ISC PDs.

## 4 Experimental Section

### Device Fabrication

An n‐type germanium sheet with a resistivity ranging from 0.1 to 6 Ω cm was used as the substrate for device fabrication. Silicon oxide was deposited on the substrate by PECVD, patterned to form windows using photolithography, and source‐drain (the thickness of Cr/Au is 5 nm/50 nm) was patterned and deposited by thermal evaporation using photolithography. Monolayer graphene was prepared by chemical vapor deposition. To transfer graphene to the substrate, a poly (methyl methacrylate)‐assisted wet transfer process was employed. After substrate drying, the graphene‐covered substrate was heated at 150 °C for 40 min to promote the attachment of graphene to Ge. Subsequently, the graphene channels were imaged by alignment lithography and oxygen etching processes, and finally the photoresist was removed with acetone. CBD was used to grow CdS on graphene, with the water temperature maintained at 82 °C and pH 10, and seven cumulative growths were performed to obtain the desired CdS thickness. Photolithography was used for patterning and the device preparation was completed by wet etching process of CdS film with 25% HCl.

### Photoelectric Response Measurement

The electrical characteristics of the devices were measured using a Keithley 4200‐SCS semiconductor analyzer. The experiments were performed using 1550 nm lasers and 450 nm lasers, and their powers were calibrated with a commercial optical power meter (Thorlabs S405C). Periodic switching of the light source was achieved by connecting the 1550 nm laser and 450 nm laser to a signal generator (Rigol DG1022U). The image information was correlated with the laser power by converting the pixel values of the grayscale image into a specific voltage sequence and loading it onto the signal generator.

### Computer Processing

For image processing, an analog convolution kernel matrix was designed to simulate the photoresponse matrix of an optoelectronic device. The simulated convolution kernels in the paper include Gauss, Roberts, and Prewitt kernels and were realized by optical modulation. The input image was encoded by pixel gray value, the gray value was extracted from the image and convolved with the optical response of the simulated convolution kernel to obtain the photocurrent, and the results of the computation were reorganized and converted to gray value to obtain the preprocessed image.

For image recognition, the grayscale images used in the experiments were first generated on a computer. The image simulation results were all calculated by the computer program. In order to improve the accuracy of CNN classification, random Gaussian noise was introduced to the original images in the training dataset to expand the number of images. A CNN of size 25 × 10 × 4 was used, which consists of an input layer, a hidden layer and an output layer, using a relu activation function and a cross‐entropy loss function. Different input images were distinguished by different output photocurrents, and the output photocurrents of each input image were distinguished by constantly updating the value of photoresponsivity during the training process, so as to realize image recognition based on photocurrents.

The process of detecting and recognizing moving vehicles in the desert to achieve motion detection includes the following steps. First, the weight matrix of NPC and PPC was loaded to extract each frame of motion from the input; next, these frames were divided into small frames of 5 × 5 pixels. Each small frame was mapped with NPC and PPC weight matrices, respectively. The mapping results of the two matrices were then summarized and the data was converted into image patterns using python.

### Statistical Analysis

In this study, photoresponse data serve as the core dataset. During preprocessing, the dark current (when the signal light was off) and the photocurrent (when the signal light was on) were extracted under single‐band or dual‐band modulation. The photoresponse current presented in this work was obtained by subtracting the dark current from the photocurrent. Each device underwent photoresponse testing at least 3–5 times, and one stable value was selected. To demonstrate the stability across devices of the same dimensions, the consistency of four devices was evaluated from the same batch. Using Microsoft Excel for statistical analysis, the dark current and photocurrent of all four devices were measured under identical conditions. The results, presented in **Tables** **2** and **3**, showed that, without modulation light, the statistical variation in dark current was within 0.63%, and the variation in photocurrent did not exceed 1.57%. Under modulation light, the variation in dark current was within 0.47%, and the photocurrent variation did not exceed 2.87%.

**Table 2:** Photoresponse of devices with the same area under signal‐band.

Device number	Dark current (I_d_)	Statistical variation of I_d_	Photocurrent (I_P_)	Statistical variation of I_P_
Device 1	4.667 mA	0.63%	0.334 mA	0.24%
Device 2	4.632 mA	0.13%	0.328 mA	1.57%
Device 3	4.615 mA	0.50%	0.336 mA	0.84%
Device 4	4.641 mA	0.06%	0.335 mA	0.54%

**Table 3:** Photoresponse of devices with the same area under dual‐band.

Device number	Dark current (I_d_)	Statistical variation of I_d_	Photocurrent (I_P_)	Statistical variation of I_P_
Device 1	4.255 mA	0.47%	0.093 mA	2.87%
Device 2	4.243 mA	0.19%	0.097 mA	1.36%
Device 3	4.217 mA	0.43%	0.095 mA	0.73%
Device 4	4.224 mA	0.26%	0.098 mA	2.40%

## Conflict of Interest

The authors declare no conflict of interest.

## Supporting information



Supporting Information

## Data Availability

Research data are not shared.
