# Supplementary material for: All‐Optical Modulation Photodetectors Based on the CdS/Graphene/Ge Sandwich Structures for Integrated Sensing‐Computing
Source: Adv Sci (Weinh). 2025 Jan 22;12(11):2413662. doi: 10.1002/advs.202413662 (PMC11923923; doi:10.1002/advs.202413662)
Supplement: Supplementary file 1 — Supporting Information [file ADVS-12-2413662-s001.docx]

Supporting Information

Title All-optical modulation photodetectors based on the CdS/graphene/Ge sandwich structures for integrated sensing-computing

Qi Yang, Jie Hu, Haozhou Li, Qing Du, Shuanglong Feng, Dong Yang, Yupeng Zhang, *, and Jun Shen*

Dr. Q. Yang, J. Hu, H. Li, S.Feng, D. Yang, Prof. J. Shen

Chongqing Institute of Green and Intelligent Technology

Chinese Academy of Sciences

Chongqing 400714, P.R. China

E-mail: shenjun@cigit.ac.cn

Q. Du, Prof. Dr. Y. Zhang

College of Electronics and Information Engineering

Shenzhen University

Shenzhen 518060, China

E-mail: ypzhang@szu.edu.cn

**Section 1:** **Supporting Figure and Its Explanation**


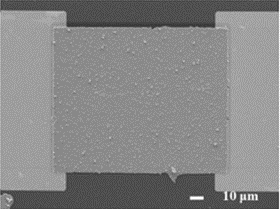


**Figure S1.** CdS/Graphene/Ge device topography.

The graphene channel size of the CdS/Graphene/Ge detector was 30 × 60 μm², and the optoelectronic performance testing environment was 80 K with a bias voltage V_ds_ of 3 V.


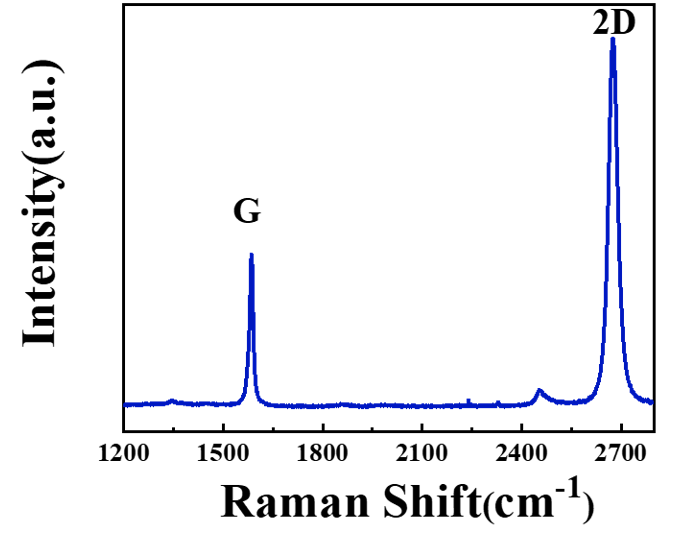


**Figure S2.** Raman spectrum of graphene, with a 2D/G ratio greater than 2, indicating high-quality graphene grown and transferred by CVD.

We characterized the Ge/Graphene/CdS device with Raman spectroscopy and absorption spectroscopy to elucidate the physical properties of the device materials and heterojunctions. Raman spectroscopy of the graphene revealed a 2D/G peak ratio greater than 2, indicating high-quality monolayer graphene grown by CVD and transferred by a wet method, as shown in **Figure** S2.

To ensure that both wavelength signals are transported through the graphene channel while minimizing crosstalk between the two wavelengths, we first prepared an electrical isolation layer between the electrodes and Ge using sputtered SiO_2_, and employed a lift-off process to create silicon oxide windows between the source and drain electrodes to ensure the formation of a heterojunction between graphene and Ge, preventing current from being transported solely through Ge. Additionally, we optimized the CdS thickness by growing CdS through 7 cycles of the chemical bath deposition (CBD) method, enhancing the absorption of 450 nm light and reducing the transmission of front-incident 450 nm light to Ge, thus mitigating signal crosstalk. The CdS absorption at 450 nm reached 96%, while the 450 μm thick Ge we used exhibited approximately 48% absorption at near-infrared 1550 nm, as shown in **Figure** S3. TEM measurements revealed that the cumulative thickness of CdS grown over 7 cycles was approximately 725 nm, as shown in **Figure** S4.


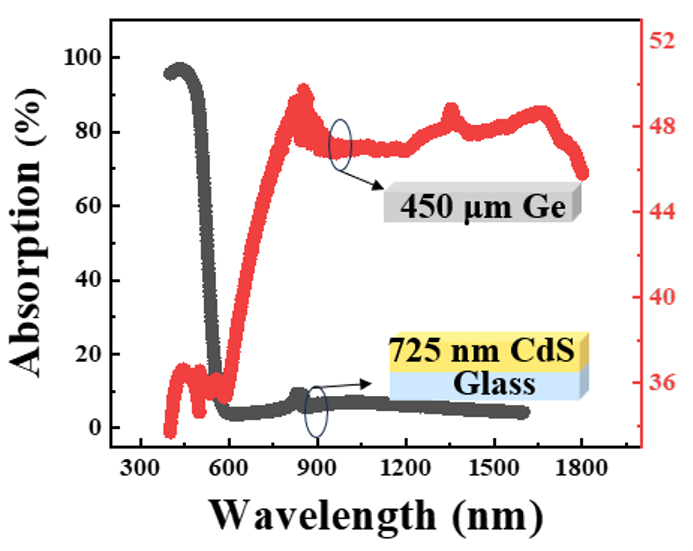


**Figure S3.** Absorptivity of approximately 725 nm thick CdS grown on K9 glass substrate by CBD, and absorptivity of 450 μm thick Ge.


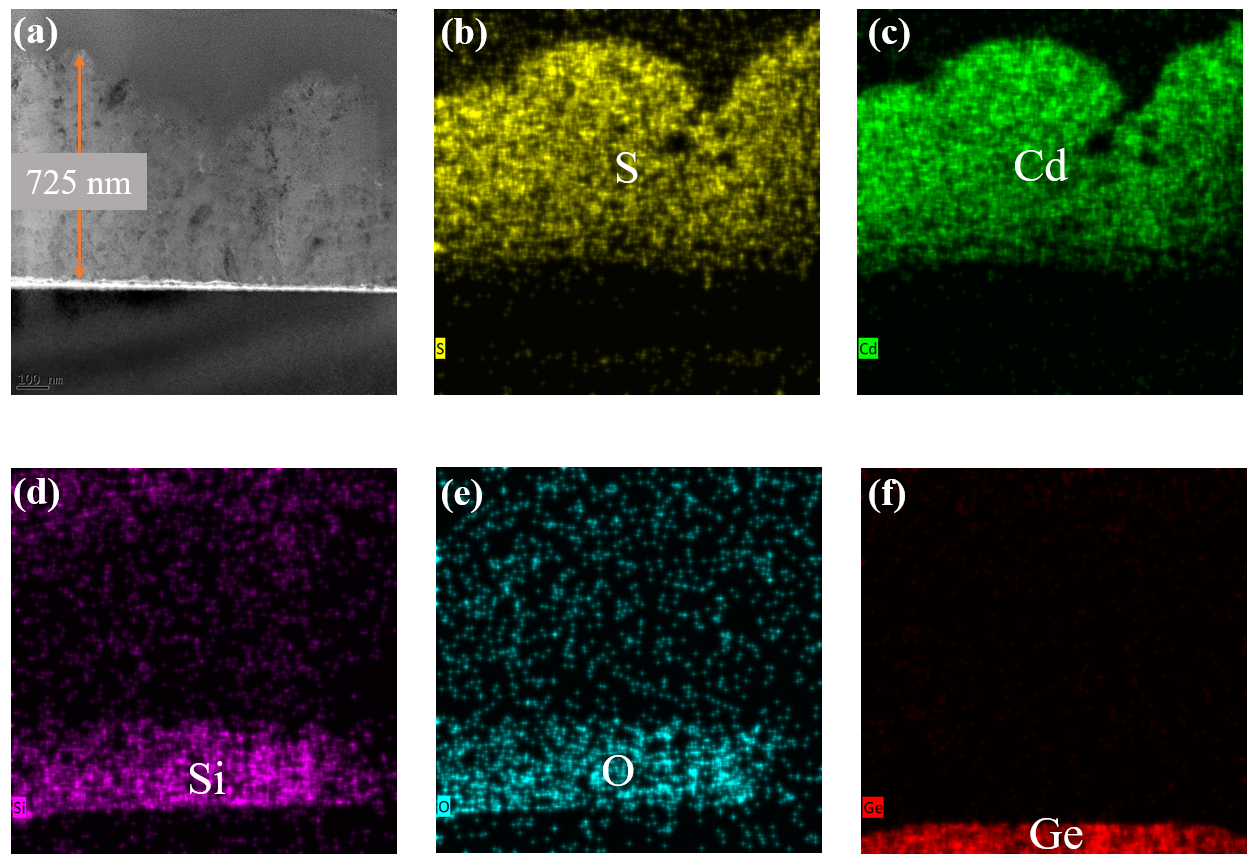


**Figure S4.** **(a)** Thickness of the CdS layer in the device cross-section tested using TEM； **(b) ~ (f)** The elemental composition information of each layer including the SiO_2_ isolation layer.

To directly observe the final morphology of the optimized heterojunction interface, we used FIB to cut and TEM to image the interface morphology of the two heterojunctions, as shown in **Figure** 1e. EDS characterization was performed to determine the elemental composition of each layer. The TEM cross-sectional images demonstrate the formation of high-quality Ge/graphene and CdS/graphene heterojunctions through van der Waals forces. Raman characterization of graphene and the thickness and absorbance characterizations of different materials collectively validate our conclusions, as detailed in the supplementary materials and **Figures** S2-S4.


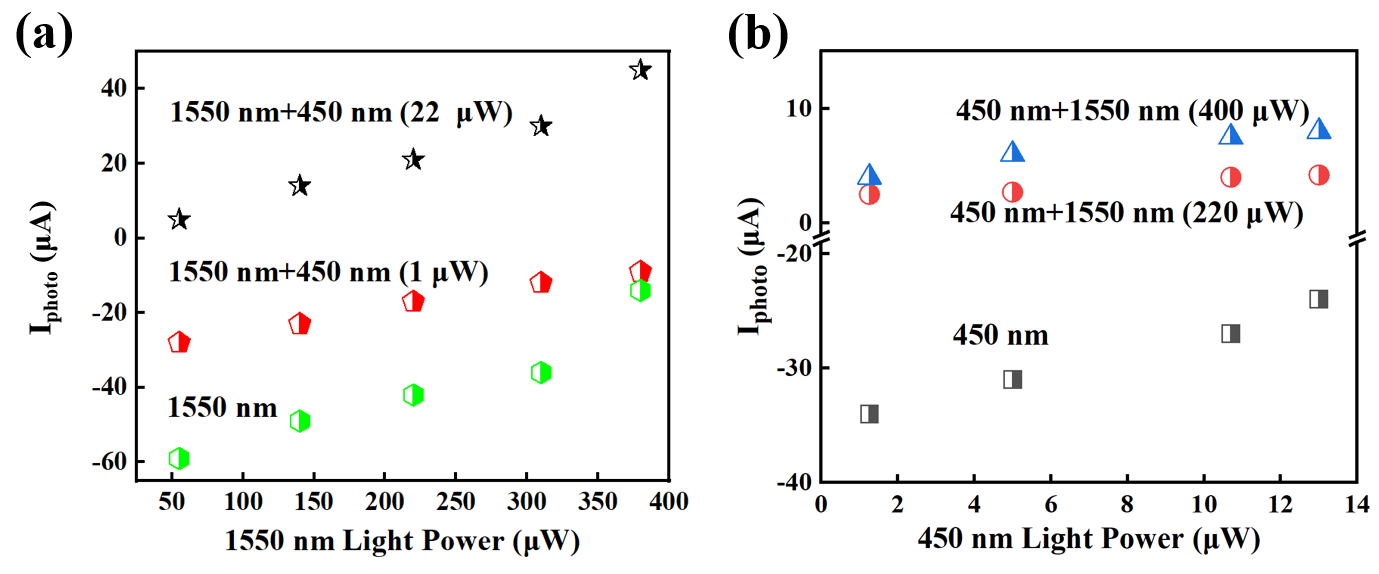


**Figure S5.** (a) Photocurrent response of the device under different power combinations of 450 nm signal light and 1550 nm modulation light. (b) Photocurrent response under different power combinations of 1550 nm signal light and 450 nm modulation light.

In our study, we quantified the variations in photocurrent across different power modulations of varying wavelength bands, as depicted in Figure 5Sa~b. Under single-wavelength laser irradiation at 450 nm or 1550 nm, a persistent Negative Photoconductance (NPC) phenomenon is observed, irrespective of changes in laser power. The absolute value of photocurrent diminishes with increasing light power, signifying a dynamic equilibrium between photogenerated carrier generation and recombination within the graphene channel under single-wavelength illumination. Prior to reaching this equilibrium, regardless of the laser power, when the CdS/Graphene or Ge/Graphene heterojunction absorbs light, generating electron-hole pairs, electrons are injected into the graphene channel, causing an elevation in the graphene's Fermi level until equilibrium is achieved, at which point the Fermi level stabilizes below the Dirac point.

When light from two different wavelength bands is simultaneously incident, the device demonstrates negative photoconductance at lower power combinations and positive photoconductance at higher power combinations, illustrating the capability to switch between these conductance states. For instance, augmenting the power of 450 nm modulated light atop the 1550 nm signal light disrupts the single-laser dynamic equilibrium. When a low total power (~1 μW) of 450 nm modulated light is applied initially, increasing the power of the 1550 nm signal light up to 389 μW still results in NPC, with the incremental value plateauing and aligning with the negative photoconductance state observed in darkness. This suggests a threshold for the number of photogenerated carriers entering the graphene channel under low-power 450 nm modulated light. Increasing the 450 nm modulated light power to 22 μW transitions the response to Positive Photoconductance (PPC), with photocurrent rising concomitantly with light power, as shown in **Figure 5S a**. Conversely, reversing the roles of the 1550 nm and 450 nm lights, with 450 nm as the signal light, as illustrated in **Figure 5S b**, results in a direct shift from negative to positive photoconductance with increasing 1550 nm modulated light power.


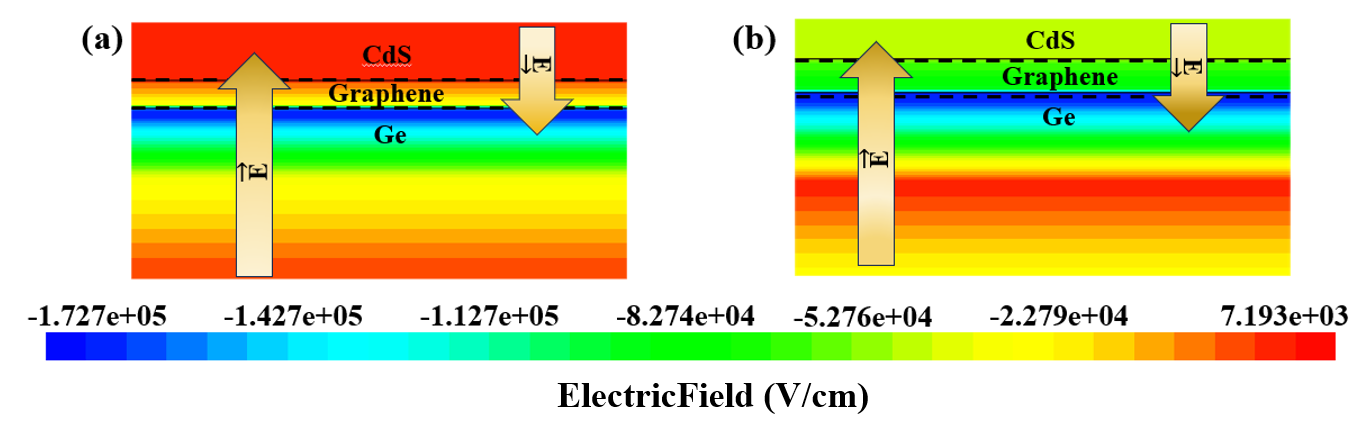


**Figure S6.** The device's electric field distribution under illumination:(a) Electric field distribution in the device under 450 nm light; (b) Electric field distribution in the device under 1550 nm light.

**Figure** 1f presents a schematic diagram of the graphene Fermi level changes when 450 nm is used as the signal light and 1550 nm as the modulation light. In the absence of illumination, the graphene is p-doped, with the Fermi level *E_f_* _0_ below the Dirac point. Upon incidence of 450 nm violet light, electrons are injected into the graphene, raising the Fermi level to above the Dirac point, reaching *E_f_* _1_, resulting in NPC. When low-power 1550 nm modulation light is added, more electrons are injected into the graphene, further raising the Fermi level to *E_f_*_2_. At this point, the photoresponse remains negative, as the photoconductance is still negative due to the Fermi level being below the symmetric point. With an increase in the power of the modulation light to a high value, the Fermi level rises further to *E_f_* _3_, surpassing the symmetric position *E_f_* _0_, resulting in PPC.

In order to further understand the mechanism of light modulation generated by CdS /Gr/ Ge dual-band devices, we conducted Sentaurus-TCAD simulation, and obtained the simulation results of electric field distribution inside the device under illumination conditions. According to the electric field distribution diagram, the direction of electric field at the CdS /Gr interface was from graphene to cadmium sulfide. At the Gr/ Ge interface, the electric field direction is from graphene to germanium, and the photogenerated electrons will also be from germanium to graphene for electron doping. The simulation results further support our interpretation of the change of Fermi energy level under light conditions.


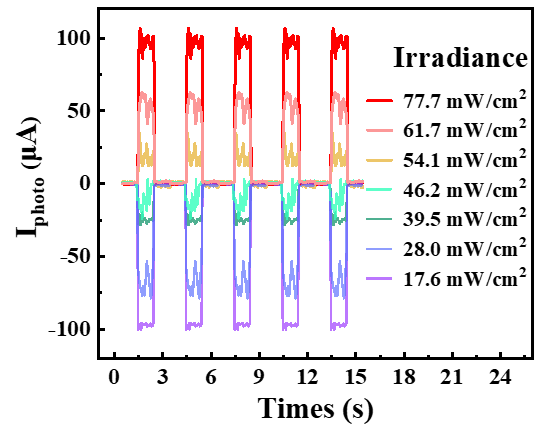


**Figure S7.** Variation of photocurrent with increasing 1550 nm modulation light power when 450 nm visible light is used as the signal light.

When the 450 nm signal light with a power density of 1.7 mW/cm² is modulated by 1550 nm infrared light with a power density of 17.6 mW/cm², the device exhibits negative photoconductance response. When the modulation power density of the 1550 nm light is increased to 77.7 mW/cm², the device shows positive photoconductance response. This indicates a significant conversion process from NPC to PPC as the modulation light power increases, as illustrated in Figure S7.


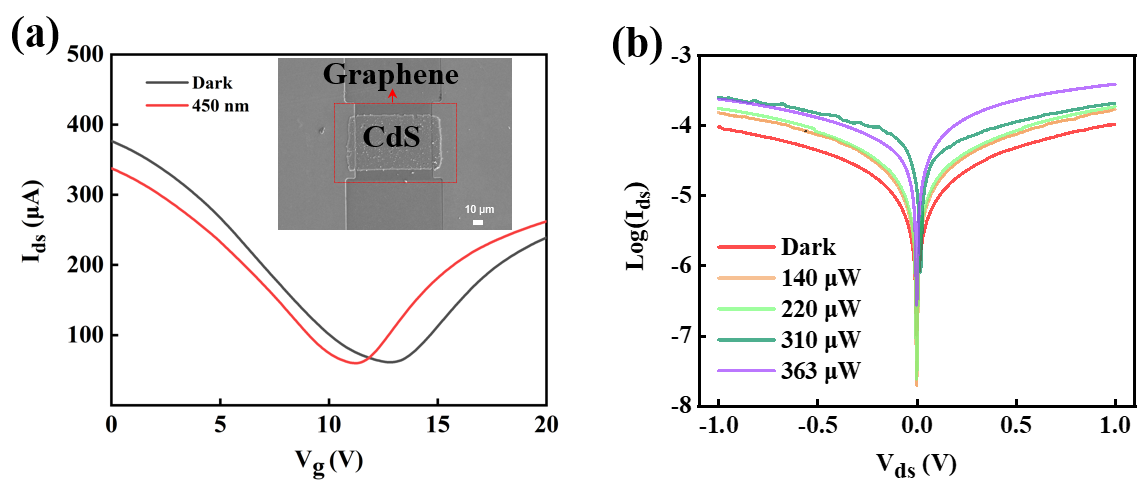


Figure S8. (a) Transfer curve of graphene/CdS devices on a Si/SiO₂ substrate, showing the light-modulated shift in the Dirac point of graphene under visible light irradiation. (b) I-V curves of the device under different 1550 nm laser powers.

To demonstrate that the injection of photogenerated carriers from the Ge/Graphene and CdS/Graphene heterojunctions into the graphene channel shifts the graphene Dirac point, we fabricated a CdS/Graphene device with back-gate modulation on a Si/SiO₂ substrate, as shown in the insert figure of **Figure** S8a. The channel transconductance of the graphene FET was tested under gate voltage modulation, as shown in **Figure** S8a, characterizing the transconductance inflection point of the CdS/Graphene channel before and after 450 nm illumination. The inflection point appeared at a positive gate voltage, indicating p-doping of the graphene. Upon illumination, the graphene Dirac point shifted to a negative gate voltage, indicating electron injection from CdS into graphene. The typical I-V curve in **Figure** S8b shows good ohmic contact characteristics and demonstrates the bipolar behavior of the graphene photodetector.


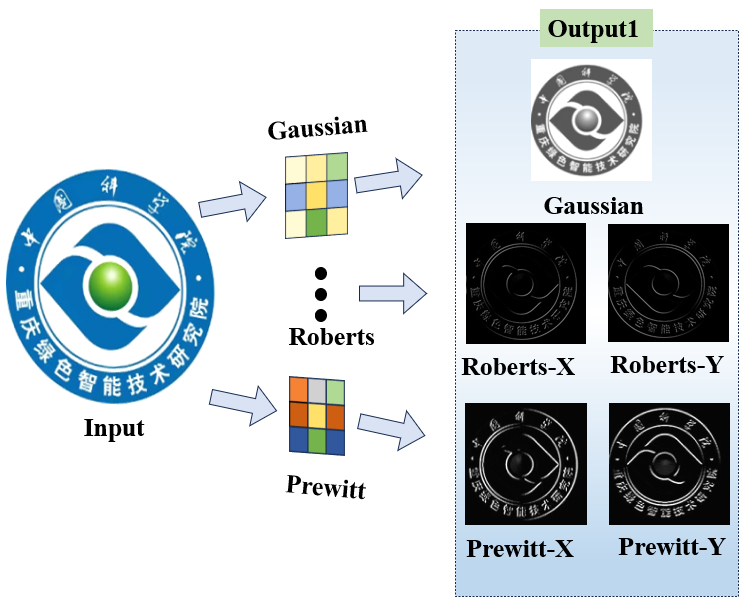


**Figure S9.** The results under Gaussian convolution kernel and Prewitt convolution kernel. The result of logo processing of Chongqing Institute of Green and Intelligent Technology of Chinese Academy of Sciences under different convolution cores.

The experimental results are simulated by a Gaussian kernel matrix with a 3×3 array simulated by our independent device. The image preprocessing operation of the Roberts operator is simulated by a 2×2 array, where individual devices write specific optical response states to different types of matrices$\left( \begin{matrix} -1 & 0 \\ 0 & 1 \end{matrix} \right)$and$\left( \begin{matrix} 0 & -1 \\ 1 & 0 \end{matrix} \right)$ are used for edge detection along the x and y axes. The image processing operation of the Prewitt operator is simulated by a 3×3 array, where individual devices write specific optical response states, and different types of matrices $\left( \begin{matrix} -1 & 0 & 1 \\ -1 & 0 & 1 \\ -1 & 0 & 1 \end{matrix} \right)$and$\left( \begin{matrix} 1 & 1 & 1 \\ 0 & 0 & 0 \\ -1 & -1 & -1 \end{matrix} \right)$ are used for edge detection along the x and y axes. In Roberts and Prewitt operators, "-1" represents the negative optical response state and "1" represents the positive optical response state.


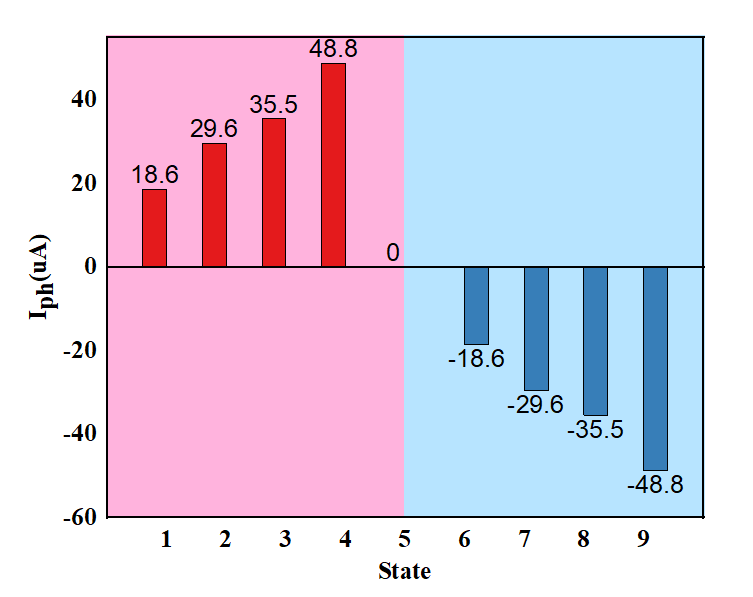


**Figure S10.** Histogram statistics of 9 photoresponse states

According to the measured photocurrent （I_photo_=I_on_-I_off_）under eight different 1550 nm modulation light power densities shown in **Figure** S10, and using the responsivity calculation formula:

$R=\frac{I_{photo}}{P\cdot\frac{S_{D}}{S_{P}}}$ (1)

where $I_{photo}$ is the photocurrent, *P* is the light power, *S_D_* is the device area, and $S_{P}$ is the light spot area (approximately 0.785 mm²). When 450 nm light is used as the signal light and 1550 nm laser as the modulation light, changing the modulation light power results in a transition of photoconductance from negative to positive.


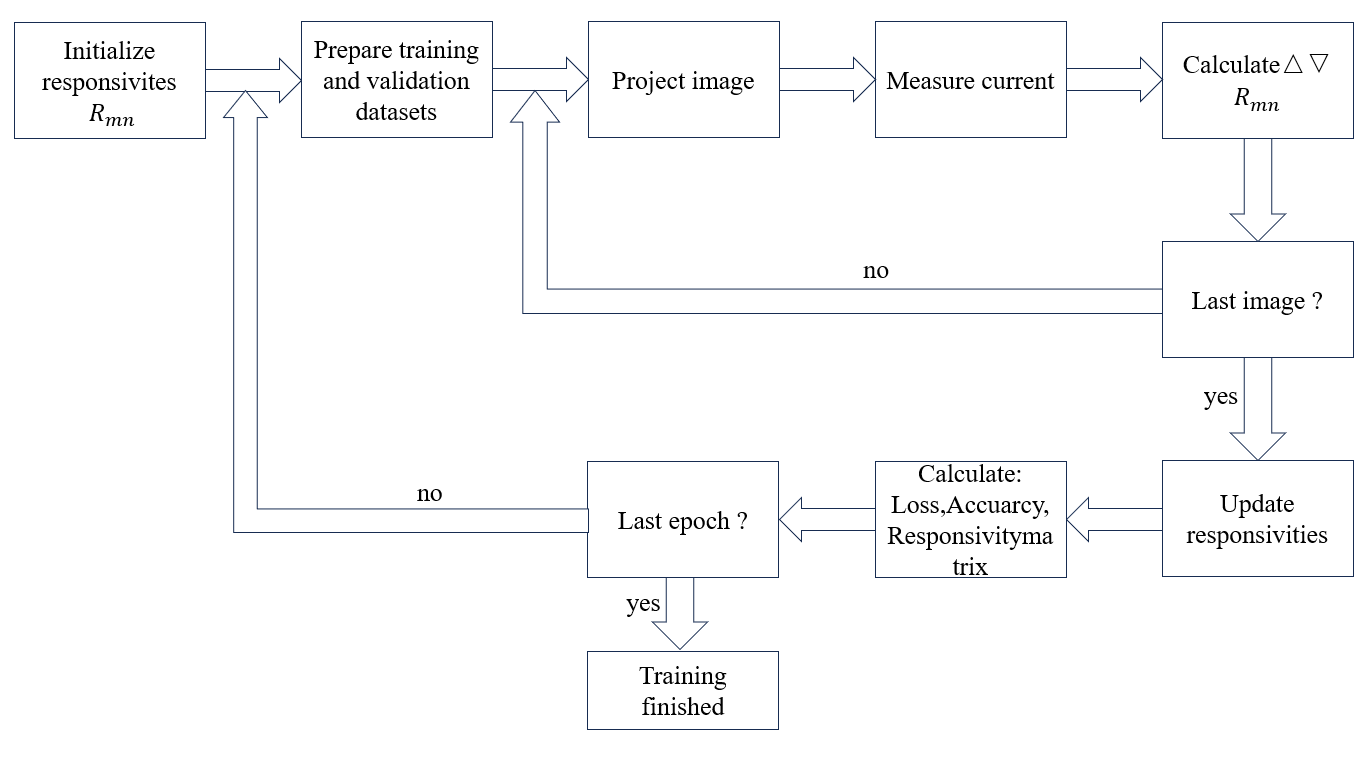


**Figure S11.** Flow chart of the training algorithm.

As shown in **Figure 3a** is the pre-training process, the training data set is used to train, the range of optical responsiveness is set based on the performance of the detector, and a neural network is built according to the classification number of probe pixels and input images of the pixelated photodetector with multi-pixels. The neural network is trained and verified by using multiple input images. The neural network after training and verification is obtained. The neural network after training and verification includes weight information and can output category information of the input image. The training adopts convolutional neural network, which includes input layer, hidden layer and output layer. Relu activation function and softmax function are selected as loss function. The value of *R* is constantly updated by backpropagation algorithm to improve the recognition accuracy, and the R weight value obtained every time in the formula is extracted to adjust the optical responsivity matrix to the state suitable for a specific input image. This process involves adjusting the optical responsivity matrix to minimize the error between the output current and the expected characteristic current. The *R* weight value extracted after training is assigned to the detector array through the adjustment device. After the assigned value, the detector receives the input image, outputs the specific current, and realizes the image recognition through the feature current. The specific current output is determined by the optical responsiveness of each unit in the detector array, thus realizing the feature recognition of the input image.

**Section 2: Theoretical Explanation of Response Time Modulation**

The slow rise time in heterojunctions formed between graphene and other materials is attributed to carrier capture and release processes caused by adsorbates and interface defects at the heterojunction interface. However, when both wavelengths of light are simultaneously incident, the 1550 nm light excites a certain number of photogenerated carriers that pre-fill the defect states at the interface, accelerating the transient response speed upon 450 nm light incidence. Additionally, the modulation light alters the built-in electric field between CdS/Graphene and Ge/Graphene, accelerating the injection of photogenerated carriers into the graphene channel, thereby enhancing the rise time response speed and effectively improving the slow response issue of the photogating effect. The fall time is not significantly affected by the modulation light, as the addition of 450 nm light does not improve the release process of trapped photogenerated carriers from interface defects. When the 1550 nm signal light is used as the modulation light, there is no observed optimization of the response time. The likely reason for this is the severe attenuation of the 450 nm laser's output power due to the fiber limitations used in this study, resulting in a maximum peak power of the 450 nm laser being less than 30 μW, which is insufficient to generate enough carriers to fill the defect states at the graphene van der Waals heterojunction interface, hence no enhancement in the response speed of the 1550 nm signal light was observed.

According to our understanding, a more detailed schematic diagram of the mechanism explanation was provided, as shown in **Figure S12**.


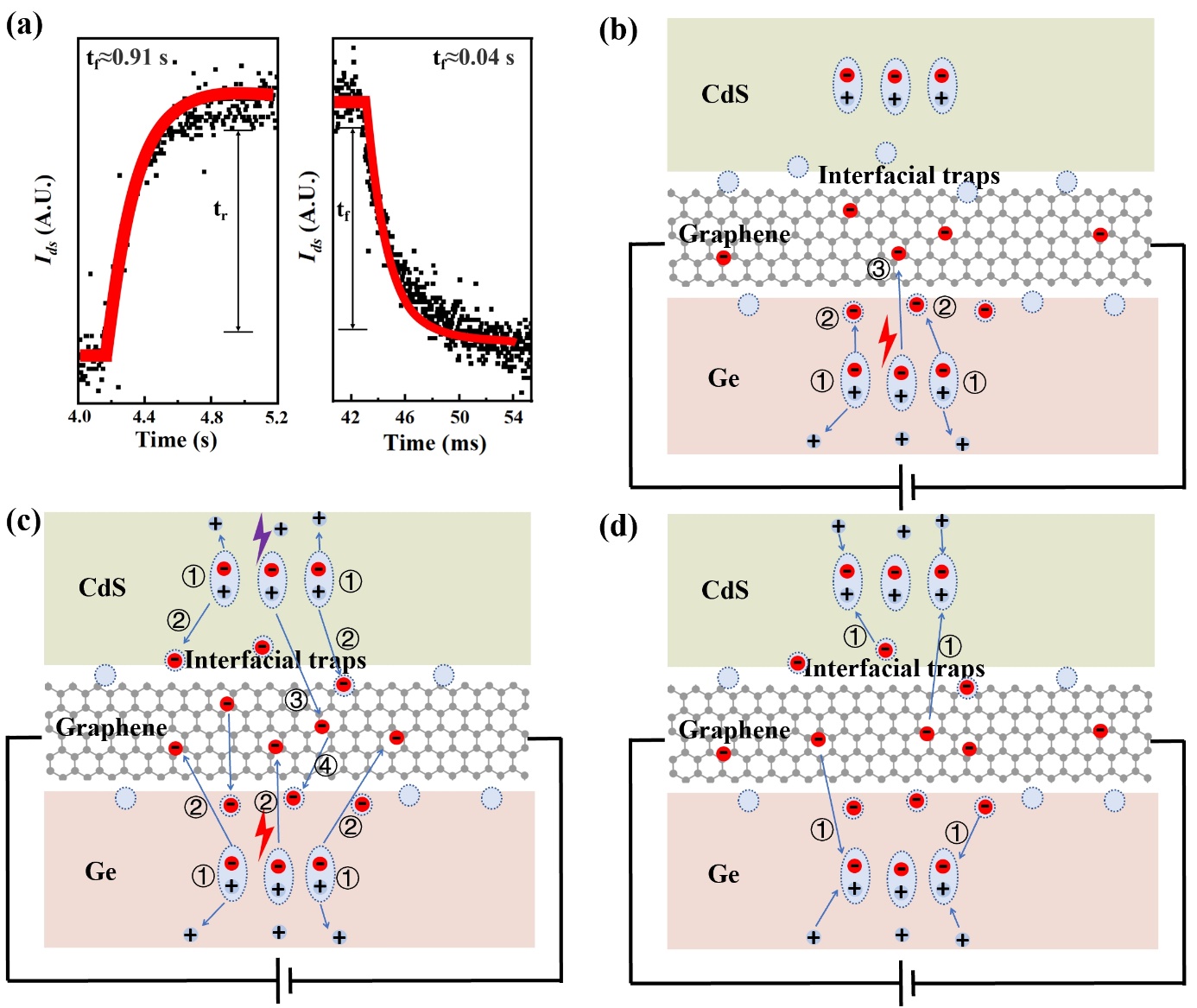


**Figure S12.** Through varying incident light conditions, the generation and transport of charge carriers are elucidated to explain the mechanism of response time modulation. (a) Response time under 1550 nm illumination without 450 nm modulation light.(b) Carrier transport mechanism of the device under 1550 nm illumination.(c) Carrier transport mechanism of the device under 1550 nm light switching with 450 nm modulation light.(d) Carrier transport mechanism of the device when all light sources are turned off.

Corresponding to **Figure S12**a, **Figure S12**b illustrates the case where only 1550 nm laser is present. Under these conditions, the photo-generated carriers induced by the 1550 nm laser underwent a three-step process: ① Generation of photo-generated carrier pairs.② Filling of defect states at the interface.③ After the defect states at the interface were fully occupied, carriers were injected into graphene, forming a photocurrent under the influence of an external voltage.

When a 450 nm modulation light was added, the carrier generation and transport process, as depicted in **Figure S12**c, became more complex. Since the light was incident from the top, CdS first absorbed the 450 nm laser, while the unabsorbed 1550 nm light penetrated to the underlying Ge substrate. For the CdS/Graphene interface, the carrier transport process consisted of four main steps:① Absorption of 450 nm light by CdS to generate photo-generated carrier pairs.② Filling of defect states at the CdS/Graphene interface by the photo-generated carriers.③ After the defect states were fully filled, carriers were injected into graphene, forming a photocurrent under an external voltage.④ Pre-filling of defect states at the Ge/Graphene interface by the photo-generated carriers.

Under a steady 450 nm modulation light, the defect states at the Ge/Graphene interface were pre-filled. When the 1550 nm laser was toggled, the following processes occurred: ① Ge absorbed the 1550 nm light to generate photo-generated carrier pairs. ② The carriers bypassed the already pre-filled defect states and were directly injected into graphene, forming a photocurrent under the applied voltage. This bypassing significantly enhanced carrier transport speed, thereby shortening the rising-edge response time.

When the illumination ceased, the carrier generation and transport processes, as shown in **Figure S12**d, determined the behavior. The trapped photo-generated electrons and those in the graphene channel recombined via relaxation after their lifetimes ended, determining the falling-edge response time. This process was independent of the pre-filling of defect states by the 450 nm modulation light and was governed solely by carrier lifetime and interface properties. Consequently, the falling-edge response time was unaffected by the modulation light.
